# *catena*-Poly[[zinc-μ-[2-(2-{[2-(2-hy­droxy­benzo­yl)hydrazinyl­idene]meth­yl}phen­oxy)acetato­(2–)]] monohydrate]

**DOI:** 10.1107/S1600536811043510

**Published:** 2011-10-29

**Authors:** Feihua Luo, Li Yang, Ping Zhang, Dan Liu

**Affiliations:** a Department of Chemistry and Chemical Engineering, Sichuan University of Arts and Science, Dazhou, Sichuan 635000, People’s Republic of China

## Abstract

In the title compound, {[Zn(C_16_H_12_N_2_O_5_)]·H_2_O}_*n*_, the unique Zn^II^ ion is coordinated in a distorted square-pyramidal environment by three O atoms and one N atom from a symmetry-unique ligand. A symmetry-related ligand provides an O atom from a carboxyl­ate group to complete the coordination in the apical site and generate a one-dimensional polymer parallel to [010]. In addition to an intra­molecular O—H⋯N hydrogen bond, inter­molecular O—H⋯O and weak C—H⋯O hydrogen bonds are observed within the one-dimensional structure.

## Related literature

For background information on zinc(II) carboxyl­ate compounds, see: Suen *et al.* (2002 ▶). For general information on the structures of carboxyl­ate and hydrazone compounds, see: Wu *et al.* (2007 ▶); Luo *et al.* (2010 ▶).
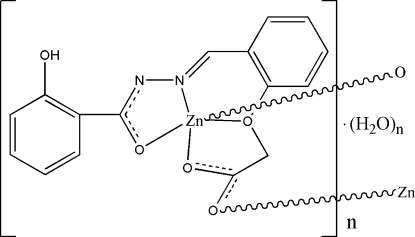


## Experimental

### 

#### Crystal data

[Zn(C_16_H_12_N_2_O_5_)]·H_2_O
*M* *_r_* = 395.66
Monoclinic, 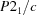

*a* = 14.730 (2) Å
*b* = 5.4063 (8) Å
*c* = 20.983 (3) Å
β = 106.620 (2)°
*V* = 1601.2 (4) Å^3^
*Z* = 4
Mo *K*α radiation
μ = 1.57 mm^−1^
*T* = 298 K
0.16 × 0.12 × 0.10 mm

#### Data collection

Bruker SMART CCD diffractometer
9785 measured reflections
3132 independent reflections
2764 reflections with *I* > 2σ(*I*)
*R*_int_ = 0.092

#### Refinement

*R*[*F* ^2^ > 2σ(*F* ^2^)] = 0.042
*wR*(*F* ^2^) = 0.109
*S* = 1.06
3132 reflections
230 parameters
H atoms treated by a mixture of independent and constrained refinement
Δρ_max_ = 0.48 e Å^−3^
Δρ_min_ = −0.51 e Å^−3^

### 

Data collection: *SMART* (Bruker, 2001 ▶); cell refinement: *SAINT* (Bruker, 2001 ▶); data reduction: *SAINT*; program(s) used to solve structure: *SHELXS97* (Sheldrick, 2008 ▶); program(s) used to refine structure: *SHELXL97* (Sheldrick, 2008 ▶); molecular graphics: *PLATON* (Spek, 2009 ▶); software used to prepare material for publication: *SHELXTL* (Sheldrick, 2008 ▶).

## Supplementary Material

Crystal structure: contains datablock(s) I, global. DOI: 10.1107/S1600536811043510/lh5346sup1.cif

Structure factors: contains datablock(s) I. DOI: 10.1107/S1600536811043510/lh5346Isup2.hkl

Additional supplementary materials:  crystallographic information; 3D view; checkCIF report

## Figures and Tables

**Table 1:** Hydrogen-bond geometry (Å, °)

*D*—H⋯*A*	*D*—H	H⋯*A*	*D*⋯*A*	*D*—H⋯*A*
O2—H20⋯N1	0.75 (4)	1.92 (4)	2.583 (3)	148 (4)
O6—H60*A*⋯O1^i^	0.84	2.22	3.056 (4)	179
C8—H8⋯O2^ii^	0.93	2.41	3.316 (4)	164

Symmetry codes: (i) 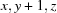; (ii) 
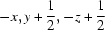
.

**Fig. 1.**
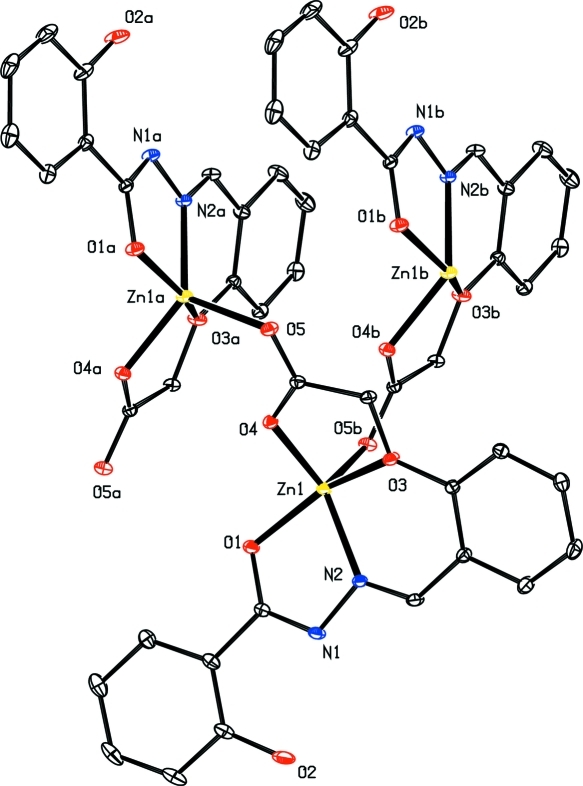
The molecular structure of with displacement ellipsoids drawn at the 30% probability level [symmetry codes: (a) -x+1, y-1/2, -z+1/2; (b) -x+1, y+1/2, -z+1/2]. H atoms are not shown.

**Fig. 2.**
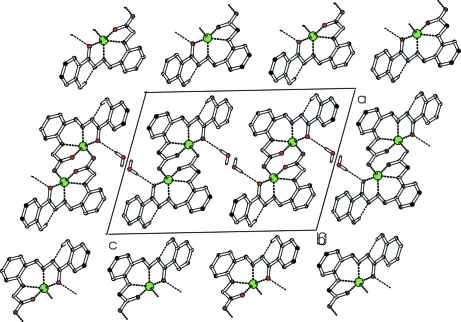
Part of the crystal structure with hydrogen bonds drawn as dashed lines. Only H atoms involved in hydrogen bonds are shown.

## supplementary crystallographic information

### Comment

Zn(II) carboxylates, especially those with nitrogen donor ligands, have been the subject of numerous investigations (Suen *et al.*, 2002). Different coordination modes of carboxylate groups can form mononuclear and polynuclear structures. Hydrazone with carboxylate groups can also form mononuclear and polynuclear structures in different conditions (Wu *et al.*, 2007; Luo *et al.*, 2010). Herein we report the synthesis and crystal structure of the title compound.

Part of the one-dimensional structure is shown in Fig. 1. The unique Zn^II^ ion is coordinated in a distorted square-pyramidal environment by three O atoms and one N atom from a symmetry unique ligand. A symmetry related ligand provides an O atom from a carboxylate group to complete the coordination in the apical site and generate a one-dimensional polymer parallel to [010] (Fig 2). In addition to an intramolecular O—H···N hydrogen bond, intermolecular O—H···O and weak C—H···O hydrogen bonds are observed within the one

### Experimental

The hydrazone ligand was synthesized according to the literature procedure (Luo *et al.*, 2010). Zinc(II) acetate monohydrate (1 mmol) was dissolved in methanol (15 ml), to which a solution of the ligand (2.5 mmol) in dimethylformamide (15 ml) was added. The mixture was stirred for 3 h at room temperature. An light-yellow solution was obtained, the solution was filtered and allowed to stand at room temperature for three weeks, where upon colorless block-shaped crystals were obtained.

### Refinement

All H atoms, except for H2O were placed in idealized positions and allowed to ride on their parent atoms, with O—H = 0.84 Å (water), C—H = 0.93-0.97Å and U_iso_=1.2–1.5 U_eq_(C,O). The hydroxy H atom (H2O) was refined independently with an isotropic displacement parameter.

### Crystal data

**Table:**

[Zn(C_16_H_12_N_2_O_5_)]·H_2_O	*F*(000) = 808
*M**_r_* = 395.66	*D*_x_ = 1.641 Mg m^−^^3^
Monoclinic, *P*2_1_/*c*	Mo *K*α radiation, λ = 0.71073 Å
Hall symbol: -P 2ybc	Cell parameters from 3978 reflections
*a* = 14.730 (2) Å	θ = 2.8–27.4°
*b* = 5.4063 (8) Å	µ = 1.57 mm^−^^1^
*c* = 20.983 (3) Å	*T* = 298 K
β = 106.620 (2)°	Block, colorless
*V* = 1601.2 (4) Å^3^	0.16 × 0.12 × 0.10 mm
*Z* = 4	

### Data collection

**Table:**

Bruker SMART CCD diffractometer	2764 reflections with *I* > 2σ(*I*)
Radiation source: fine-focus sealed tube	*R*_int_ = 0.092
graphite	θ_max_ = 26.0°, θ_min_ = 2.0°
φ and ω scans	*h* = −16→18
9785 measured reflections	*k* = −6→6
3132 independent reflections	*l* = −25→25

### Refinement

**Table:**

Refinement on *F*^2^	Primary atom site location: structure-invariant direct methods
Least-squares matrix: full	Secondary atom site location: difference Fourier map
*R*[*F*^2^ > 2σ(*F*^2^)] = 0.042	Hydrogen site location: inferred from neighbouring sites
*wR*(*F*^2^) = 0.109	H atoms treated by a mixture of independent and constrained refinement
*S* = 1.06	*w* = 1/[σ^2^(*F*_o_^2^) + (0.0552*P*)^2^ + 0.0104*P*] where *P* = (*F*_o_^2^ + 2*F*_c_^2^)/3
3132 reflections	(Δ/σ)_max_ = 0.001
230 parameters	Δρ_max_ = 0.48 e Å^−^^3^
0 restraints	Δρ_min_ = −0.51 e Å^−^^3^

### Special details

**Table:**

Geometry. All e.s.d.'s (except the e.s.d. in the dihedral angle between two l.s. planes) are estimated using the full covariance matrix. The cell e.s.d.'s are taken into account individually in the estimation of e.s.d.'s in distances, angles and torsion angles; correlations between e.s.d.'s in cell parameters are only used when they are defined by crystal symmetry. An approximate (isotropic) treatment of cell e.s.d.'s is used for estimating e.s.d.'s involving l.s. planes.
Refinement. Refinement of *F*^2^ against ALL reflections. The weighted *R*-factor *wR* and goodness of fit *S* are based on *F*^2^, conventional *R*-factors *R* are based on *F*, with *F* set to zero for negative *F*^2^. The threshold expression of *F*^2^ > σ(*F*^2^) is used only for calculating *R*-factors(gt) *etc*. and is not relevant to the choice of reflections for refinement. *R*-factors based on *F*^2^ are statistically about twice as large as those based on *F*, and *R*- factors based on ALL data will be even larger.

### Fractional atomic coordinates and isotropic or equivalent isotropic displacement parameters (Å^2^)

**Table:**

	*x*	*y*	*z*	*U*_iso_*/*U*_eq_	
Zn1	0.37549 (2)	0.04095 (6)	0.273648 (16)	0.03342 (14)	
O1	0.33632 (13)	−0.1912 (4)	0.33490 (10)	0.0408 (5)	
O2	0.04795 (17)	−0.1639 (6)	0.32481 (16)	0.0713 (8)	
O3	0.35695 (13)	0.1914 (4)	0.16947 (9)	0.0381 (5)	
O4	0.45124 (12)	−0.1779 (3)	0.23378 (9)	0.0347 (4)	
O5	0.54232 (13)	−0.2163 (4)	0.16671 (9)	0.0401 (5)	
N1	0.18798 (16)	−0.0372 (4)	0.28074 (13)	0.0380 (6)	
N2	0.23483 (15)	0.1069 (4)	0.24511 (11)	0.0320 (5)	
C1	0.2011 (2)	−0.3599 (5)	0.35985 (14)	0.0396 (7)	
C2	0.1069 (2)	−0.3391 (7)	0.35924 (17)	0.0522 (8)	
C3	0.0705 (3)	−0.5039 (8)	0.3972 (2)	0.0722 (12)	
H3	0.0084	−0.4860	0.3988	0.087*	
C4	0.1252 (3)	−0.6905 (7)	0.43192 (19)	0.0711 (12)	
H4	0.0998	−0.8000	0.4564	0.085*	
C5	0.2174 (3)	−0.7179 (6)	0.43094 (17)	0.0624 (10)	
H5	0.2541	−0.8471	0.4541	0.075*	
C6	0.2553 (3)	−0.5528 (5)	0.39543 (16)	0.0475 (8)	
H6	0.3181	−0.5705	0.3952	0.057*	
C7	0.24582 (19)	−0.1871 (5)	0.32341 (13)	0.0334 (6)	
C8	0.1840 (2)	0.2634 (5)	0.20485 (14)	0.0402 (7)	
H8	0.1218	0.2832	0.2061	0.048*	
C9	0.2143 (2)	0.4136 (5)	0.15722 (14)	0.0364 (6)	
C10	0.29665 (18)	0.3766 (5)	0.13731 (13)	0.0317 (6)	
C11	0.3143 (2)	0.5182 (5)	0.08720 (15)	0.0384 (7)	
H11	0.3689	0.4913	0.0743	0.046*	
C12	0.2508 (2)	0.6998 (5)	0.05635 (15)	0.0460 (7)	
H12	0.2627	0.7940	0.0225	0.055*	
C13	0.1696 (2)	0.7423 (6)	0.07554 (16)	0.0536 (8)	
H13	0.1275	0.8663	0.0552	0.064*	
C14	0.1519 (2)	0.5995 (6)	0.12493 (17)	0.0516 (8)	
H14	0.0969	0.6274	0.1372	0.062*	
C15	0.42785 (19)	0.1002 (5)	0.14144 (14)	0.0343 (6)	
H15A	0.4731	0.2296	0.1406	0.041*	
H15B	0.3992	0.0441	0.0962	0.041*	
C16	0.47695 (18)	−0.1127 (5)	0.18446 (13)	0.0332 (6)	
O6	0.4589 (3)	0.7641 (10)	0.47903 (19)	0.171 (2)	
H60A	0.4249	0.7787	0.4396	0.256*	
H60B	0.5164	0.7397	0.4823	0.256*	
H20	0.074 (3)	−0.090 (7)	0.305 (2)	0.067 (13)*	

### Atomic displacement parameters (Å^2^)

**Table:**

	*U*^11^	*U*^22^	*U*^33^	*U*^12^	*U*^13^	*U*^23^
Zn1	0.0246 (2)	0.0419 (2)	0.0363 (2)	0.00089 (12)	0.01278 (15)	0.00451 (13)
O1	0.0301 (10)	0.0488 (12)	0.0463 (12)	0.0011 (9)	0.0154 (9)	0.0140 (9)
O2	0.0348 (13)	0.093 (2)	0.094 (2)	−0.0094 (14)	0.0308 (14)	0.0222 (17)
O3	0.0306 (10)	0.0517 (12)	0.0363 (11)	0.0114 (9)	0.0163 (8)	0.0117 (9)
O4	0.0331 (10)	0.0378 (10)	0.0368 (10)	0.0055 (8)	0.0157 (8)	0.0039 (8)
O5	0.0315 (10)	0.0508 (11)	0.0411 (11)	0.0072 (9)	0.0153 (9)	0.0016 (9)
N1	0.0270 (12)	0.0492 (14)	0.0417 (14)	−0.0039 (10)	0.0160 (11)	0.0052 (11)
N2	0.0240 (11)	0.0389 (12)	0.0354 (13)	0.0020 (9)	0.0123 (10)	0.0045 (10)
C1	0.0453 (17)	0.0440 (16)	0.0323 (15)	−0.0146 (14)	0.0156 (13)	−0.0054 (12)
C2	0.0429 (18)	0.061 (2)	0.055 (2)	−0.0190 (17)	0.0189 (16)	0.0034 (17)
C3	0.067 (3)	0.085 (3)	0.076 (3)	−0.038 (2)	0.038 (2)	−0.004 (2)
C4	0.100 (3)	0.064 (2)	0.058 (2)	−0.043 (2)	0.037 (2)	−0.0004 (19)
C5	0.093 (3)	0.052 (2)	0.0418 (19)	−0.019 (2)	0.0199 (19)	0.0033 (15)
C6	0.059 (2)	0.0452 (17)	0.0401 (18)	−0.0089 (15)	0.0163 (16)	−0.0020 (13)
C7	0.0319 (14)	0.0365 (14)	0.0339 (14)	−0.0066 (11)	0.0130 (12)	−0.0046 (11)
C8	0.0257 (14)	0.0531 (17)	0.0448 (17)	0.0032 (12)	0.0147 (13)	0.0049 (14)
C9	0.0345 (15)	0.0409 (15)	0.0342 (15)	0.0071 (12)	0.0104 (12)	0.0046 (12)
C10	0.0282 (13)	0.0345 (13)	0.0308 (14)	0.0006 (11)	0.0056 (11)	−0.0004 (11)
C11	0.0381 (16)	0.0416 (15)	0.0359 (16)	−0.0031 (12)	0.0112 (13)	0.0004 (12)
C12	0.057 (2)	0.0418 (16)	0.0381 (16)	−0.0013 (14)	0.0127 (15)	0.0060 (13)
C13	0.063 (2)	0.0492 (18)	0.0487 (19)	0.0229 (16)	0.0155 (17)	0.0140 (15)
C14	0.0488 (19)	0.0548 (19)	0.055 (2)	0.0195 (16)	0.0202 (16)	0.0116 (16)
C15	0.0279 (14)	0.0438 (15)	0.0337 (15)	0.0032 (12)	0.0127 (12)	0.0008 (12)
C16	0.0275 (14)	0.0370 (14)	0.0354 (15)	−0.0026 (11)	0.0094 (12)	−0.0041 (12)
O6	0.097 (3)	0.327 (7)	0.087 (3)	−0.054 (4)	0.023 (2)	−0.067 (4)

### Geometric parameters (Å, °)

**Table:**

Zn1—O4	1.9694 (17)	C4—C5	1.372 (6)
Zn1—O5^i^	1.9717 (19)	C4—H4	0.9300
Zn1—O1	1.9958 (18)	C5—C6	1.379 (4)
Zn1—N2	2.017 (2)	C5—H5	0.9300
Zn1—O3	2.2743 (18)	C6—H6	0.9300
O1—C7	1.285 (3)	C8—C9	1.454 (4)
O2—C2	1.347 (4)	C8—H8	0.9300
O2—H20	0.75 (4)	C9—C14	1.400 (4)
O3—C10	1.380 (3)	C9—C10	1.406 (4)
O3—C15	1.425 (3)	C10—C11	1.385 (4)
O4—C16	1.250 (3)	C11—C12	1.382 (4)
O5—C16	1.259 (3)	C11—H11	0.9300
O5—Zn1^ii^	1.9717 (19)	C12—C13	1.387 (4)
N1—C7	1.322 (4)	C12—H12	0.9300
N1—N2	1.392 (3)	C13—C14	1.376 (4)
N2—C8	1.276 (3)	C13—H13	0.9300
C1—C2	1.389 (4)	C14—H14	0.9300
C1—C6	1.393 (4)	C15—C16	1.513 (4)
C1—C7	1.476 (4)	C15—H15A	0.9700
C2—C3	1.400 (5)	C15—H15B	0.9700
C3—C4	1.365 (6)	O6—H60A	0.8400
C3—H3	0.9300	O6—H60B	0.8400
			
O4—Zn1—O5^i^	110.48 (8)	C5—C6—H6	119.5
O4—Zn1—O1	101.48 (8)	C1—C6—H6	119.5
O5^i^—Zn1—O1	104.35 (8)	O1—C7—N1	124.8 (2)
O4—Zn1—N2	129.69 (9)	O1—C7—C1	119.0 (2)
O5^i^—Zn1—N2	117.57 (9)	N1—C7—C1	116.3 (2)
O1—Zn1—N2	80.84 (8)	N2—C8—C9	126.0 (2)
O4—Zn1—O3	74.54 (7)	N2—C8—H8	117.0
O5^i^—Zn1—O3	104.79 (7)	C9—C8—H8	117.0
O1—Zn1—O3	150.04 (8)	C14—C9—C10	117.5 (3)
N2—Zn1—O3	79.67 (8)	C14—C9—C8	116.5 (3)
C7—O1—Zn1	110.12 (16)	C10—C9—C8	125.9 (2)
C2—O2—H20	109 (3)	O3—C10—C11	122.6 (2)
C10—O3—C15	119.63 (19)	O3—C10—C9	116.6 (2)
C10—O3—Zn1	127.81 (15)	C11—C10—C9	120.9 (3)
C15—O3—Zn1	111.85 (14)	C12—C11—C10	119.9 (3)
C16—O4—Zn1	121.68 (17)	C12—C11—H11	120.0
C16—O5—Zn1^ii^	119.40 (17)	C10—C11—H11	120.0
C7—N1—N2	112.6 (2)	C11—C12—C13	120.4 (3)
C8—N2—N1	116.1 (2)	C11—C12—H12	119.8
C8—N2—Zn1	132.25 (18)	C13—C12—H12	119.8
N1—N2—Zn1	111.50 (16)	C14—C13—C12	119.4 (3)
C2—C1—C6	118.9 (3)	C14—C13—H13	120.3
C2—C1—C7	122.5 (3)	C12—C13—H13	120.3
C6—C1—C7	118.6 (3)	C13—C14—C9	121.9 (3)
O2—C2—C1	123.4 (3)	C13—C14—H14	119.1
O2—C2—C3	117.4 (3)	C9—C14—H14	119.1
C1—C2—C3	119.2 (4)	O3—C15—C16	107.4 (2)
C4—C3—C2	120.7 (4)	O3—C15—H15A	110.2
C4—C3—H3	119.7	C16—C15—H15A	110.2
C2—C3—H3	119.7	O3—C15—H15B	110.2
C3—C4—C5	120.6 (3)	C16—C15—H15B	110.2
C3—C4—H4	119.7	H15A—C15—H15B	108.5
C5—C4—H4	119.7	O4—C16—O5	123.9 (3)
C4—C5—C6	119.6 (4)	O4—C16—C15	120.1 (2)
C4—C5—H5	120.2	O5—C16—C15	115.9 (2)
C6—C5—H5	120.2	H60A—O6—H60B	113.4
C5—C6—C1	121.0 (3)		
			
O4—Zn1—O1—C7	−126.34 (18)	C7—C1—C6—C5	179.3 (3)
O5^i^—Zn1—O1—C7	118.79 (18)	Zn1—O1—C7—N1	−4.7 (3)
N2—Zn1—O1—C7	2.52 (18)	Zn1—O1—C7—C1	175.39 (18)
O3—Zn1—O1—C7	−47.4 (3)	N2—N1—C7—O1	4.5 (4)
O4—Zn1—O3—C10	172.7 (2)	N2—N1—C7—C1	−175.7 (2)
O5^i^—Zn1—O3—C10	−79.7 (2)	C2—C1—C7—O1	168.5 (3)
O1—Zn1—O3—C10	86.5 (2)	C6—C1—C7—O1	−12.4 (4)
N2—Zn1—O3—C10	36.3 (2)	C2—C1—C7—N1	−11.4 (4)
O4—Zn1—O3—C15	−17.10 (17)	C6—C1—C7—N1	167.7 (3)
O5^i^—Zn1—O3—C15	90.50 (17)	N1—N2—C8—C9	−172.7 (3)
O1—Zn1—O3—C15	−103.3 (2)	Zn1—N2—C8—C9	12.9 (5)
N2—Zn1—O3—C15	−153.49 (18)	N2—C8—C9—C14	−170.7 (3)
O5^i^—Zn1—O4—C16	−81.2 (2)	N2—C8—C9—C10	14.4 (5)
O1—Zn1—O4—C16	168.6 (2)	C15—O3—C10—C11	−15.1 (4)
N2—Zn1—O4—C16	81.0 (2)	Zn1—O3—C10—C11	154.5 (2)
O3—Zn1—O4—C16	19.1 (2)	C15—O3—C10—C9	164.0 (2)
C7—N1—N2—C8	−177.3 (2)	Zn1—O3—C10—C9	−26.5 (3)
C7—N1—N2—Zn1	−1.6 (3)	C14—C9—C10—O3	−179.6 (3)
O4—Zn1—N2—C8	−88.4 (3)	C8—C9—C10—O3	−4.7 (4)
O5^i^—Zn1—N2—C8	72.7 (3)	C14—C9—C10—C11	−0.5 (4)
O1—Zn1—N2—C8	174.2 (3)	C8—C9—C10—C11	174.3 (3)
O3—Zn1—N2—C8	−28.7 (3)	O3—C10—C11—C12	179.4 (2)
O4—Zn1—N2—N1	96.90 (19)	C9—C10—C11—C12	0.4 (4)
O5^i^—Zn1—N2—N1	−101.94 (17)	C10—C11—C12—C13	0.4 (4)
O1—Zn1—N2—N1	−0.47 (17)	C11—C12—C13—C14	−1.0 (5)
O3—Zn1—N2—N1	156.67 (18)	C12—C13—C14—C9	0.8 (5)
C6—C1—C2—O2	−178.0 (3)	C10—C9—C14—C13	−0.1 (5)
C7—C1—C2—O2	1.2 (5)	C8—C9—C14—C13	−175.4 (3)
C6—C1—C2—C3	3.5 (5)	C10—O3—C15—C16	−175.6 (2)
C7—C1—C2—C3	−177.4 (3)	Zn1—O3—C15—C16	13.3 (2)
O2—C2—C3—C4	178.1 (4)	Zn1—O4—C16—O5	162.7 (2)
C1—C2—C3—C4	−3.2 (6)	Zn1—O4—C16—C15	−18.2 (3)
C2—C3—C4—C5	0.9 (6)	Zn1^ii^—O5—C16—O4	−10.5 (4)
C3—C4—C5—C6	1.1 (6)	Zn1^ii^—O5—C16—C15	170.31 (18)
C4—C5—C6—C1	−0.7 (5)	O3—C15—C16—O4	0.8 (3)
C2—C1—C6—C5	−1.5 (5)	O3—C15—C16—O5	180.0 (2)

Symmetry codes: (i) −*x*+1, *y*+1/2, −*z*+1/2; (ii) −*x*+1, *y*−1/2, −*z*+1/2.

### Hydrogen-bond geometry (Å, °)

**Table:**

*D*—H···*A*	*D*—H	H···*A*	*D*···*A*	*D*—H···*A*
O2—H20···N1	0.75 (4)	1.92 (4)	2.583 (3)	148 (4)
O6—H60A···O1^iii^	0.84	2.22	3.056 (4)	179.
C8—H8···O2^iv^	0.93	2.41	3.316 (4)	164.

Symmetry codes: (iii) *x*, *y*+1, *z*; (iv) −*x*, *y*+1/2, −*z*+1/2.
